# Quest for safe and feasible isolation of superior vena cava by pulsed-field ablation: are we there yet?

**DOI:** 10.1093/europace/euae159

**Published:** 2024-06-14

**Authors:** Moussa Mansour, Sanghamitra Mohanty, Andrea Natale

**Affiliations:** Electrophysiology, Harvard Medical School, Boston, MA, USA; Texas Cardiac Arrhythmia Institute, St. David’s Medical Center, 3000 N. I-35, Suite 720, Austin, TX 78705, USA; Texas Cardiac Arrhythmia Institute, St. David’s Medical Center, 3000 N. I-35, Suite 720, Austin, TX 78705, USA; Interventional Electrophysiology, Scripps Clinic, 10666 N Torrey Pines Rd, La Jolla, CA 92037, USA; Department of Internal Medicine, Metro Health Medical Center, Case Western Reserve University School of Medicine, 2500 Metrohealth Dr, Cleveland, OH 44109, USA; Department of Biomedicine and Prevention, Division of Cardiology, University of Tor Vergata, Via Montpellier, 1, Rome 00133, Italy

## Abstract

**Graphical Abstract:**
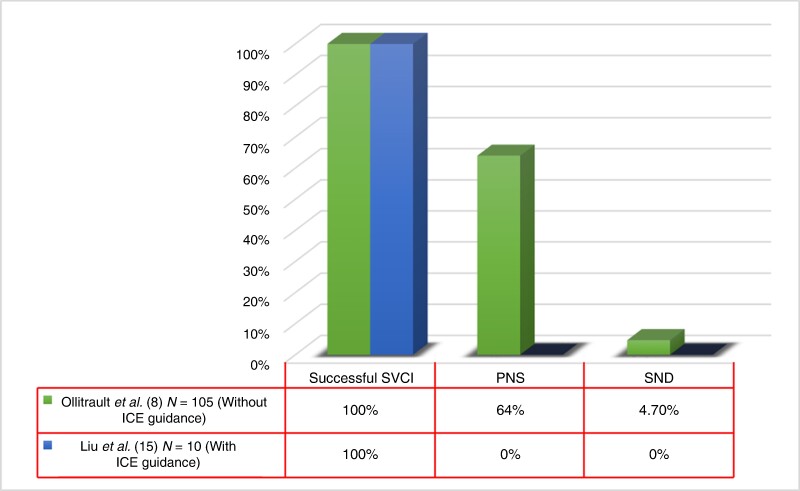
Bar diagram shows ablation outcomes in patients undergoing PFA-based SVCI with vs. without ICE guidance. PNS, phrenic nerve stunning; SND, sinus node dysfunction; ICE, intracardiac echocardiography; SVCI, superior vena cava isolation.

Bar diagram shows ablation outcomes in patients undergoing PFA-based SVCI with vs. without ICE guidance. PNS, phrenic nerve stunning; SND, sinus node dysfunction; ICE, intracardiac echocardiography; SVCI, superior vena cava isolation.

**This editorial refers to ‘Superior vena cava isolation using a pentaspline pulsed-field ablation catheter: feasibility and safety in patients undergoing atrial fibrillation catheter ablation’, by P. Ollitrault *et al.*, https://doi.org/10.1093/europace/euae160.**

Superior vena cava (SVC) is one of the major non-pulmonary vein (PV) triggers of atrial fibrillation (AF). Cells retaining the pacing abilities, embedded in the myocardial sleeves that extend into the SVC, serve as foci for arrhythmogenesis.^1^ Superior vena cava musculature is also known to be independently capable of sustaining AF.^2^ Moreover, SVC cardiomyocytes are influenced by autonomic innervation, and it has been demonstrated that the adjacent aortocaval ganglion preferentially triggers SVC ectopic beats.^3^ As a result of the above, adjunctive SVC isolation (SVCI) has been performed, in addition to PVI, in certain groups of patients undergoing ablation for AF.^4–7^ The procedure, however, can be associated with complications including stenosis of the vein, phrenic nerve injury (PNI), and sinus node dysfunction (SND). Thermal ablation has been associated with 2–5% incidence of PNI,^1^ and the proximity of the phrenic nerve has been shown to prevent SVCI in 13–18% of the patients.^1^ Proposed strategies to minimize these risks include the use of intracardiac echocardiography (ICE) to define SVC–right atrium junction, low power ablation, identification of the sinus node with high-density mapping, and localization of the phrenic nerve with pacing prior to ablation.^1^

Pulsed-field ablation (PFA) is a non-thermal energy modality that uses high-voltage electric pulses to cause cell death. Therefore, it is prudent to speculate that the risk of collateral damage to the phrenic nerve and sinus node would be significantly lower with PFA compared with thermal ablation.

The current study by Ollitrault *et al*.^8^ evaluated the feasibility and safety of SVCI using PFA in patient undergoing ablation for AF. One hundred and five consecutive patients were included, 64% of whom had persistent AF, and only 5% had prior ablation. The pentaspline PFA catheter in the basket configuration was used to deliver four to eight PFA applications at the target location. Phrenic nerve pacing was performed using the ablation catheter, before and after energy delivery. Additionally, right diaphragmatic compound motor action potential was utilized for monitoring of phrenic nerve activity. The heart rate was also monitored before and after each PFA application to assess SND. The acute safety outcomes consisted of the incidence of phrenic nerve palsy (PNP) and SND. Acute PNP was defined as loss of diaphragmatic contraction at the end of the procedure and SND as complete sinus arrest during procedure and until discharge. Complete SVCI was achieved in all patients with a mean duration of workflow of 3.0 ± 1.3 min. During the procedure, 67 (64%) of the patients experienced transient phrenic nerve stunning (PNS). No evidence of PNP was recorded at the end of the procedure and at 3-month follow-up. Seven (6.7%) patients experienced SND leading to transient junctional rhythm that resolved spontaneously at the end of the procedure. No PFA-induced implantable cardioverter-defibrillator or pacemaker dysfunction occurred in the 10 patients with a cardiac implantable electronic device.

The study had several minor limitations including post-procedure evaluation of the phrenic nerve using chest X-ray only in a very small number of patients, the lack of 24-h Holter monitoring to evaluate SND, and the lack of follow-up imaging to assess SVC stenosis. Despite these shortcomings, this study is important because it represents the largest series to date to describe the safety and feasibility of SVCI using PFA, and as such, it will pave the way for future larger multicentre investigations to validate the findings.

The safety and feasibility of PFA for SVCI have been documented in previous case reports and animal studies. In a pre-clinical study, transmural SVCI was achieved using PFA without any incidence of PNI, SND, or stenosis of SVC.^9^ Ellejmi *et al*.^10^ published a case report on SVCI using PFA with no complications. Tsiachris *et al*.^11^ described the successful isolation of the SVC using PFA in two patients with defibrillator leads. In another case report, SVCI using PFA was associated with transient SND that lasted for around 30 min before recovery of normal sinus rhythm.^12^ Tao *et al*.^13^ demonstrated effective termination of arrhythmia using PFA-based SVCI without any procedural complications in a patient with paroxysmal AF and four prior ablations. Successful uncomplicated isolation of persistent left SVC using PFA has also been described in two patients.^14^

All the studies above suggest that SVCI using PFA appears to be highly feasible. However, the occurrence of transient PNS in 64% patients in the current series by Ollitrault *et al*.^8^ is not trivial and highlights the need for certain measures aiming at reducing or eliminating this incident. Localization of the phrenic nerve with pacing or imaging (i.e. ICE) is achievable in most patients that could facilitate a safer procedure (*Graphical Abstract*).^15^ Perhaps, a strategy of limiting the number of application in proximity of the phrenic nerve reduces the transient effect.

In conclusion, the data from the current series demonstrate the feasibility of SVCI using PFA, and the authors should be commended. Larger studies are required to validate the findings and design dosing strategies to minimize the transient effect on the phrenic nerve and the sinus node.

## Data Availability

No new data were generated or analyzed in support of this research.
